# Evaluation of Insecticides Susceptibility and Malaria Vector Potential of *Anopheles annularis* s.l. and *Anopheles vagus* in Assam, India

**DOI:** 10.1371/journal.pone.0151786

**Published:** 2016-03-24

**Authors:** Sunil Dhiman, Kavita Yadav, Bipul Rabha, Diganta Goswami, S. Hazarika, Varun Tyagi

**Affiliations:** Division of Medical Entomology, Defence Research Laboratory, Tezpur, Assam, 784001, India; Institut Pasteur, FRANCE

## Abstract

During the recent past, development of DDT resistance and reduction to pyrethroid susceptibility among the malaria vectors has posed a serious challenge in many Southeast Asian countries including India. Current study presents the insecticide susceptibility and knock-down data of field collected *Anopheles annularis* sensu lato and *An*. *vagus* mosquito species from endemic areas of Assam in northeast India. *Anopheles annularis* s.l. and *An*. *vagus* adult females were collected from four randomly selected sentinel sites in Orang primary health centre (OPHC) and Balipara primary health centre (BPHC) areas, and used for testing susceptibility to DDT, malathion, deltamethrin and lambda-cyhalothrin. After insecticide susceptibility tests, mosquitoes were subjected to VectorTest^™^ assay kits to detect the presence of malaria sporozoite in the mosquitoes. *An*. *annularis* s.l. was completely susceptible to deltamethrin, lambda-cyhalothrin and malathion in both the study areas. *An*. *vagus* was highly susceptible to deltamethrin in both the areas, but exhibited reduced susceptibility to lambda-cyhalothrin in BPHC. Both the species were resistant to DDT and showed very high KDT_50_ and KDT_99_ values for DDT. Probit model used to calculate the KDT_50_ and KDT_99_ values did not display normal distribution of percent knock-down with time for malathion in both the mosquito species in OPHC (p<0.05) and *An*. *vagus* in BPHC (χ^2^ = 25.3; p = 0.0), and also for deltamethrin to *An*. *vagus* in BPHC area (χ^2^ = 15.4; p = 0.004). Minimum infection rate (MIR) of *Plasmodium* sporozoite for *An*. *vagus* was 0.56 in OPHC and 0.13 in BPHC, while for *An*. *annularis* MIR was found to be 0.22 in OPHC. Resistance management strategies should be identified to delay the expansion of resistance. Testing of field caught *Anopheles* vectors from different endemic areas for the presence of malaria sporozoite may be useful to ensure their role in malaria transmission.

## Introduction

In the recent years, scaling-up of long-lasting insecticidal nets (LLINs) and to some extent indoor residual spraying (IRS) using insecticides has been a pivotal element in mosquito control strategies. However, rapid emergence and geographical spread of insecticide resistance among malaria vectors has threatens the intervention programmes in many endemic Afro-Asian countries. Only four insecticide classes, which share two modes of action have been approved by the World Health Organization (WHO) for use in mosquito control programmes [1]. Since there are limited number of insecticide groups available, the options to switch over to comparatively more effective insecticide in control operations are restricted. Considering the importance of insecticides in malaria control, regular monitoring of insecticides susceptibility among *Anopheles* vectors is essential, primarily in the regions where malaria in endemic and subsistently remains a burden to the ethnic communities [2–4].

In India, synthetic pyrethroids have been widely used in LLINs, while DDT is used for IRS in many malaria endemic regions including northeastern states of India. However, few recent studies have indicated considerable level of resistance among some well established malaria vectors against synthetic pyrethroids and DDT in different states of India [5–8]. The northeast region of India is geographically isolated and shares international frontiers on three sides with malaria affected countries like Bangladesh, Bhutan and Myanmar. In this region, although *Anopheles minimus* and *An*. *dirus* are considered as major malaria vectors [5, 9, 10], but recently the abundance of these two mosquito species has decreased [11], while *An*. *annularis* and *An*. *vagus* became increasingly important due to their high density during the peak malaria season and possible role in malaria transmission. Although both these species are primarily zoophilic, exophilic and exophagic, but also found feeding on human blood and maintaining malaria transmission in the plain areas of northeast India and adjoining Bangladesh [6, 9, 12, 13].

*Anopheles annularis* Van der Wulp, 1884 is widespread in many Asian countries and recently emerged as an important malaria vector in India and neighbouring countries [6, 10, 14, 15]. *An*. *vagus* Doenitz, 1902 is extensively recorded in malaria endemic areas of Indian sub-continent and plays important role in malaria transmission in Bangladesh, Laos and Cambodia [12, 13, 16, 17]. Previous studies have shown resistance to DDT and reduced susceptibility to deltamethrin in *An*. *annularis* [6], but none of the study has recorded the insecticide resistance status of *An*. *vagus* in northeast India. Although *An*. *vagus* is a well known malaria vector in many countries now, but in India, no study has demonstrated its potential role in malaria transmission.

The objective of the present study was to investigate the insecticide susceptibility of *An*. *annularis* s.l. and *An*. *vagus* in malaria endemic Udalguri district and Sonitpur district of Assam in northeast India. Since organochloride (OC), synthetic pyrethroids (SP) and organophosphate (OP) insecticides are used in malaria control intervention in the region, we have used DDT, deltamethrin, lambda-cyhalothrin and malathion as test insecticides in this study. Furthermore, VectorTest^™^ malaria sporozoite antigen panel assay has been used for the detection of *Plasmodium* circumsporozoite antigens in both the mosquitoes species.

## Materials and Methods

### Study area

**Fig 1:**
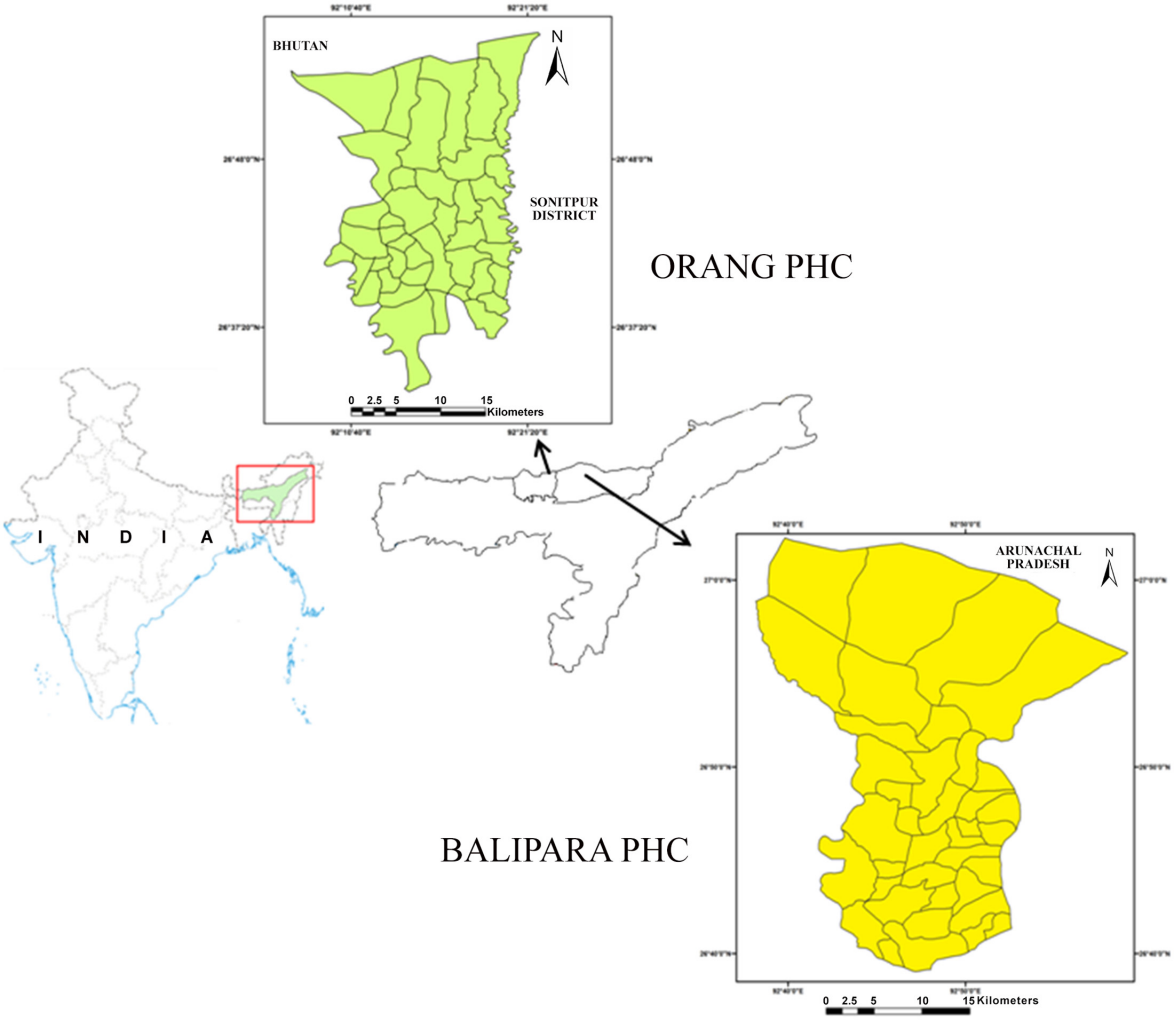
Study area map. Map showing Balipara and Orang primacy health centre (BPHC and OPHC) where mosquito collections were performed for WHO insecticide resistance test.

Current study was conducted during March 2013 to August 2013 (pre-monsoon and monsoon season) in malaria endemic Udalguri and Sonitpur districts of Assam state of northeast India. In Udalguri district, four sentinel sites each were randomly selected in Orang primary health centre (OPHC) (26° 33’–26° 56’ N to 92° 07’–92° 22’ E), while in Sonitpur district, same number of sentinel sites were chosen in Balipara primary health centre area (BPHC) (26° 41’–27° 02’ N to 92° 38’–92° 59’ E) (Fig 1). The study districts are dominated by socio-economically backward ethnic tribes engaged mainly in tea based agriculture [4]. The climate is humid with an average annual rainfall of about 2,000 mm and temperature varying between 13.5°C to 35.0°C. Study area has many small rivers, duck rearing ponds, spread of tea gardens, vast paddy fields and forests, which provide sufficient breeding habitat for mosquitoes. Both the primary health centres report high incidence of malaria annually [4,18–20]. During the study year OPHC area reported malaria parasite slide positivity rate (SPR) of 2.40 and annual parasitic index (API) of 3.76, whereas BPHC area reported SPR and API of 2.12 and 0.24 respectively. Insecticides use has been intensive with several rounds of spray per growing season due to severe damage of tea and rice by insect pests. Synthetic pyrethroids are most commonly used in agriculture, whereas the use of organophosphate and carbamate based insecticides is comparatively less common. No specific permissions were required for conducting this activity in both the study areas. We have made the collection of mosquitoes only and none of the study in this research involved the collection and use of rare/endangered/protected animal species.

### Mosquito collection, identification and resistance bioassay

Adult indoor resting mosquitoes were collected from the human houses during 0500–600 hours using suction tube and torch light. Mosquitoes were identified morphologically using standard keys used for the identification of medically important *Anopheles* mosquitoes. The study areas were subjected to a round of indoor residual spray of DDT in February 2013. Healthy and unfed adult females of *An*. *annularis* s.l. and *An*. *vagus* were exposed to World Health Organisation (WHO) insecticide pre-impregnated papers of DDT (4%), deltamethrin (0.05%), lambda-cyhalothrin (0.05%) and malathion (5%) obtained from University Sains Malaysia, Malaysia in WHO insecticide susceptibility evaluation test kits [21,22]. The control tests were performed using pre-impregnated paper with silicone oil (deltamethrin and lambda-cyhalothrin control), risella oil (DDT control) and olive oil (malathion control) along with each set of insecticide bioassay. Each time 10–15 mosquitoes were used in the test for 1 hour and cumulative knock-down was recorded after an interval of 10 minutes [6]. The mosquitoes were then transferred into the holding tube and fed on 5% sucrose solution. Mortality was recorded after a 24 hour holding period and the resistance status was defined according to WHO guidelines, which state that 98–100% mortality indicates susceptibility, 90–98% indicates the possibility of resistance that needs to be confirmed and <90% indicates resistance [23]. After the completion of each test, mosquitoes were re-identified to avoid any error and stored in labeled eppendorf tubes for malaria sporozoite antigen detection assay using VectorTest^™^ assay kit according to the standard manufacturer’s instruction.

### Malaria sporozoite antigen assay

The adult female *An*. *annularis* s.l. and *An*. *vagus* were subjected to VectorTest^™^ assay kits (Vector Test System Inc., CA) to detect the presence of malaria sporozoite in the mosquitoes. For each test a pool of 20–25 mosquitoes of a species was put into the grinding tube provided with the assay kit and homogenised in grinding solution using plastic pestle. The tests were performed according to the standard manufacturer’s instruction provided with the assay kit. The VectorTest^™^ malaria sporozoite antigen assay is a highly specific and rapid immunochromatographic test for qualitative determination of circumsporozoite antigens of *P*. *falciparum*, *P*. *vivax* 210 and *P*. *vivax* 247 malaria parasite species in infected mosquitoes [24,25].

### Data analysis

The mortality obtained in the mosquito species was corrected using Schneider-Orelli's formula [26]. Knock-down time (KDT_50_ and KDT_99_) along with slope and 95% confidence interval (CI) were determined using Ldp Line computer programme. Chi-square (χ^2^) test was used to analyse the fitment of probit, while liner regression was used to evaluate if data deviate from linearity.

## Results

### Insecticide resistance bioassay

A total of 1,566 *An*. *annularis* s.l. and 1,998 *An*. *vagus* mosquitoes were tested in the present study to determine the susceptibility against insecticides (Tables 1 and 2). As per WHO guidelines [23], the mortality ranging between 98–100% indicates susceptibility, 90–97% as tolerant for which further investigation is needed, and <90% is considered resistant where pre-emptive action is required to manage the resistance against insecticides used for malaria vector control. Based on these recommendations, *An*. *annularis* s.l. was completely susceptible to deltamethrin, lambda-cyhalothrin and malathion in both the study PHCs as the corrected mortality observed was 100% (95% CI- 97.5–100.0), 99.3% (95% CI- 96.3–99.9) and 98.7% (95% CI- 95.3–99.6) respectively in OPHC (Table 1), while 98.1% (95% CI- 95.8–99.2), 98.6% (95% CI- 94.9–99.6) and 98.9% (95% CI- 96.1–99.7) respectively for the three insecticides in BPHC area (Table 2). *An*. *annularis* s.l. from both the study areas showed complete resistance to DDT and the corrected mortality recorded was below 75.2% (95% CI- 68.2–82.1). *An*. *vagus* mosquitoes were highly susceptible to deltamethrin but exhibited considerably reduced susceptibility to lambda-cyhalothrin in both the areas. The mortality of *An*. *vagus* to DDT was recorded below 83.3% (95% CI- 95.3–99.6) in the present study which indicated a high level of resistance to DDT. Against malathion, *An*. *vagus* mosquitoes were susceptible in OPHC (corrected mortality- 99.3%; 95% CI- 96.3–99.9), while suspected to be resistant in BPHC as the corrected mortality was found to be 97.6% (95% CI- 95.4–98.8) (Tables 1 and 2).

**Table 1:** Toxicity and knock-down time of *An*. *annularis* and *An*. *vagus* in Orang primary health centre (OPHC) area.

Insecticide (N)	Mosquito species	%KD_1h_ (N)	KDT_50_ (95% CI)	KDT_99_ (95% CI)	Slope±SD	χ^2^ (p)	r	CM_24 h_ (95% CI)
Deltamethrin (150)	*An*. *annularis*	90.7 (136)	24.7 (22.9–26.5)	129.0 (108.6–160.4)	3.2±0.2	1.9 (0.7)	1	100 (97.5–100.0)
DDT (150)		41.3 (62)	99.2 (71.7–181.4)	14960.1 (3210.2–319454.1)	1.1±0.2	1.1 (0.9)	1	75.2 (68.6–82.1)
Malathion (150)		62 (93)	51.2 (43.0–73.3)	384.7 (341.7–1583.1)	2.7±0.2	14.7 (0.005)	0.9	99.3 (96.3–99.9)
L-cyhalothrin (150)		90 (135)	24.5 (22.8–26.1)	116.7 (99.6–142.2)	3.4±0.2	1.8 (0.8)	1	98.7 (95.3–99.6)
Deltamethrin (150)	*An*. *vagus*	96 (144)	22.1 (20.6–23.7)	102.4 (88.4–122.8)	3.5±0.2	8.0 (0.08)	1	98.7 (95.3–99.6)
DDT (150)		35.3 (53)	159.5 (100.2–421.3)	33625.9 (5099.1–2039497.9)	1.0±0.2	4.1 (0.3)	0.9	83.3 (76.6–88.5)
Malathion (150)		68 (102)	41.2 (34.9–50.9)	252.5 (203.4–566.9)	2.9±0.2	11.7 (0.02)	1	99.3 (96.3–99.9)
L-cyhalothrin (150)		90 (135)	25.2 (23.6–26.8)	109.1 (94.2–131.0)	3.7±0.2	2.6 (0.6)	1	97.3 (93.3–98.9)

KDT—knock-down time in minutes; CM—corrected mortality in percent; N- number, CI—confidence interval, SD—standard deviation, r—correlation coefficient

**Table 2:** Toxicity and knock-down time of *An*. *annularis* and *An*. *vagus* in Balipara primary health centre (BPHC) area.

Insecticide (N)	Mosquito species	%KD_1h_ (N)	KDT_50_ (95% CI)	KDT_99_ (95% CI)	Slope±SD	χ^2^ (p)	r	CM_24 h_ (95% CI)
Deltamethrin (272)	*An*. *annularis*	86.8 (236)	25.3 (23.9–26.7)	144.0 (124.8–170.9)	3.1±0.2	4.0 (0.9)	1	98.1 (95.8–99.2)
DDT (373)		37.3 (139)	91.4 (79.8–109.3)	1357.3 (841.5–2592.5)	2.0±0.2	3.1 (0.5)	1	65.1 (61.3–70.8)
Malathion (139)		79.9 (111)	37.7 (35.4–40.3)	170.8 (139.6–222.6)	3.5±0.3	6.6 (0.2)	1	98.6 (94.9–99.6)
L-cyhalothrin (182)		80.8 (147)	31.8 (29.6–34.1)	231.3 (182.8–313.1)	2.7±0.2	4.7 (0.3)	1	98.9 (96.1–99.7)
Deltamethrin (424)	*An*. *vagus*	98.1 (416)	20.5 (18.1–22.7)	85.8 (76.4–105.9)	3.7±0.1	15.4 (0.004)	1	99.1 (97.6–99.6)
DDT (326)		36.5 (119)	105.6 (87.6–136.8)	2831.6 (1444.8–7437.6)	1.6±0.1	3.6 (0.5)	1	70.0 (65.1–74.9)
Malathion (335)		90.7 (304)	38.6 (34.8–42.5)	91.9 (86.1–115.6)	6.2±0.3	25.3 (0.0)	1	97.6 (95.4–98.8)
L-cyhalothrin (313)		83.1 (260)	31.1 (29.8–32.5)	147.2 (129.7–171.1)	3.4±0.2	0.3 (1.0)	1	88.6 (84.9–91.9)

KDT—knock-down time in minutes; CM—corrected mortality in percent; N- number, CI—confidence interval, SD—standard deviation, r—correlation coefficient

### Knock-down effect

**Fig 2:**
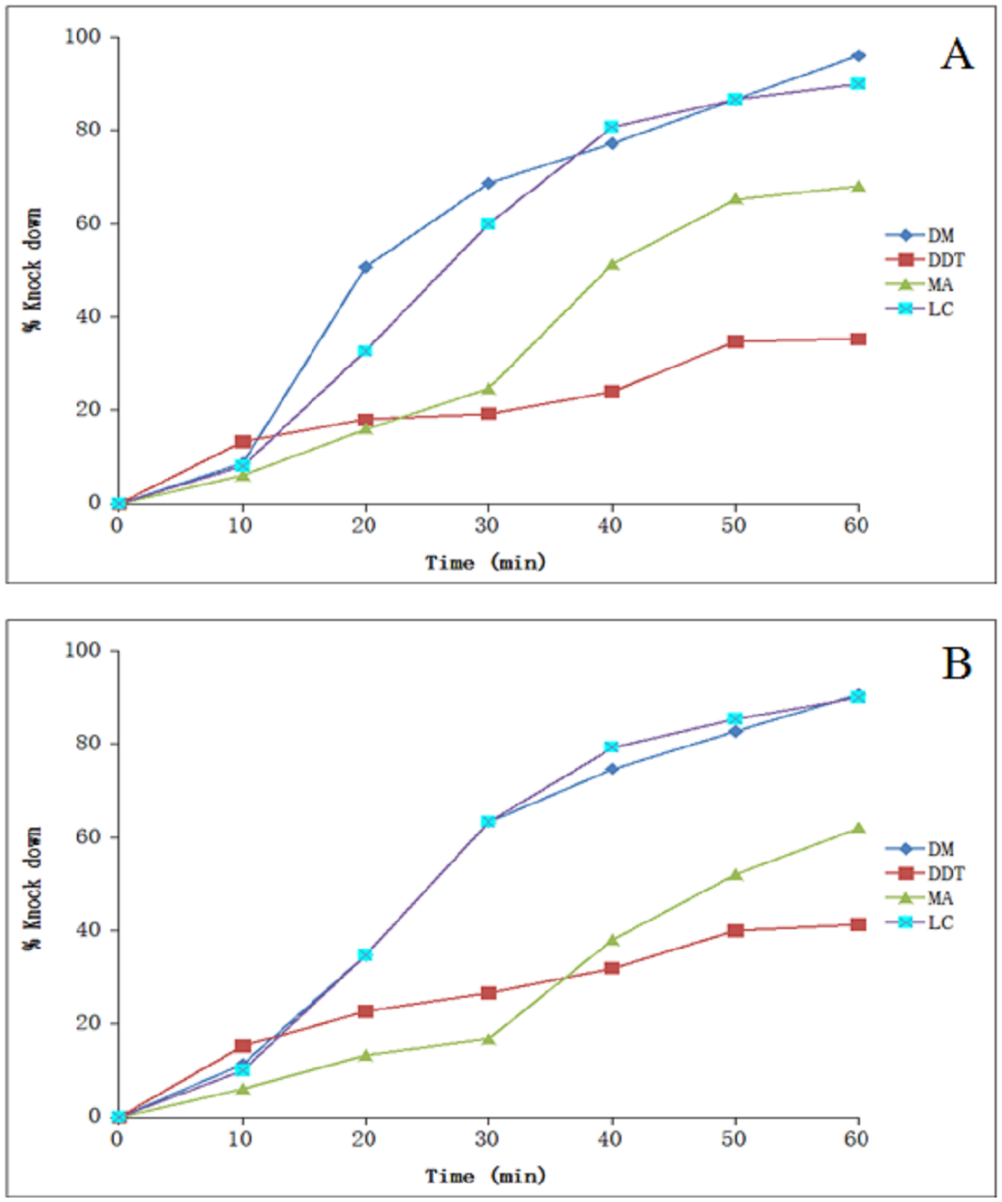
Knock-down rate for different insecticides during 1 hour of exposure in Orang primary health centre (OPHC) area. *An*. *vagus* (A), *An*. *annularis* (B). DM- deltamethrin, MA- malathion, LC- lambda-cyhalothrin.

**Fig 3:**
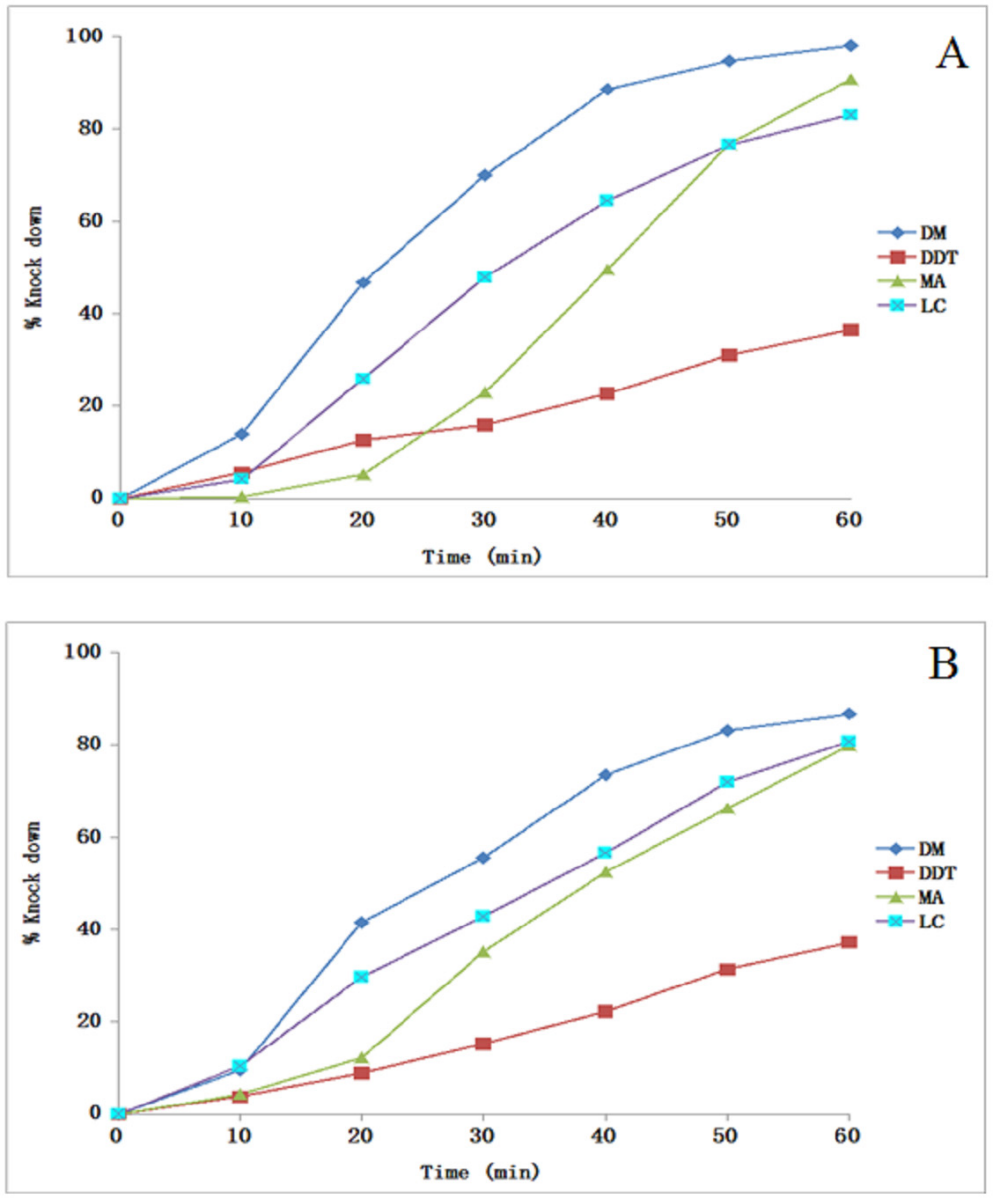
Knock-down rate for different insecticides during 1 hour of exposure in Balipara primary health centre (BPHC) area. *An*. *vagus* (A), *An*. *annularis* (B). DM- deltamethrin, MA- malathion, LC- lambda-cyhalothrin.

The knock-down effect of four insecticides determined against *An*. *vagus* and *An*. *annularis* s.l. in OPHC and BPHC over an exposure time period of one hour has been shown in Tables 1 and 2, whereas the percent knock-down achieved in both the locations has been depicted in Fig 2 (S1 Table) and Fig 3 (S2 Table) respectively. In *An*. *annularis* s.l., the KDT_50_ ranged from 24.7 to 25.3 minutes, while KDT_99_ ranged from 129.0 to 144.0 minutes during the study. Among all the tested insecticides, least knock-down percent of both the mosquito species (range—35.3–41.3%) was recorded for DDT, whereas highest knock-down percent ranging from 86.8 to 98.1 was observed for deltamethrin within one hour of exposure time. Both the mosquito species displayed very high KDT_50_ and KDT_99_ values for DDT in both the study locations. The KDT_50_ values of malathion and lambda-cyhalothrin ranged from 37.7 to 51.2 and 24.5 to 31.8 minutes respectively. Presently the probit model used to calculate the KDT_50_ and KDT_99_ values displayed normal distribution of percent knock-down with time for all the insecticides except malathion for both the mosquito species in OPHC (p<0.05) and *An*. *vagus* in BPHC (χ^2^ = 25.3; p = 0.0), and also for deltamethrin exposure to *An*. *vagus* in BPHC area (χ^2^ = 15.4; p = 0.004).

### Malaria sporozoite antigen assay

In the present study, a total of 59 pools (N = 1,340) of female *An*. *vagus* and 39 pools (N = 780) of *An*. *annularis* s.l were tested for the presence of *Plasmodium* sporozoite antigen (S3 Table). For *An*. *vagus*, 3 pools were found positive in OPHC, whereas 1 pool was positive in BPHC area. Minimum infection rate (MIR) of *Plasmodium* sporozoite for *An*. *vagus* was found to be 0.56 in OPHC and 0.13 in BPHC. On the other hand only 1 pool was found positive for *An*. *annularis* in OPHC with a MIR of 0.22 and pool positive rate of 4.35 (Table 3). All the tested mosquito pools found positive for *Plasmodium* sporozoites corresponded to malaria parasite *Plasmodium falciparum*.

**Table 3:** *Plasmodium* sporozoite detection using VectorTest^™^ panel assay.

Species	Location	Pool (n)	N	Positive	MIR	PPR
*An*. *vagus*	Orang	27 (20)	540	3	0.56	11.11
	Balipara	32 (25)	800	1	0.13	3.13
*An*. *annularis*	Orang	23 (20)	460	1	0.22	4.35
	Balipara	16 (20)	320	0	0.00	0.00
Total		98	2120	5	0.24	5.10

N—total number tested; n—number in each pool; MIR—minimum infection rate; PPR—pool positive rate

## Discussion

Presently, WHO insecticide bioassays were performed on *An*. *annularis* s.l. and *An*. *vagus* mosquitoes to assess their susceptibility to DDT, deltamethrin, malathion and lambda-cyhalothrin in two endemic districts of Assam in northeast India. Different level of susceptibility to the tested insecticides has been observed in the study. WHO recommends the use of 2–3 days old female mosquitoes for insecticide bioassay, however currently field collected mosquitoes representing natural age-structured populations were tested to determine the resistance status. Hence there was a mix of mosquitoes of different age, which probably produced higher mortality than expected by using young mosquitoes. Previous studies have reported that as compared to the young mosquitoes, the level of detoxifying enzymes, namely GST and monooxygenase often decreases with age, leading to an increase in the insecticide susceptibility level [17, 27, 28].

The results demonstrated that both the mosquito species displayed a high level of biological resistance to DDT as the corrected mortality ranged from 83.3% (95% CI = 76.6–88.5) to as low as 65.1% (95% CI = 61.3–70.8) during the study. Although DDT is extensively used in public health programmes, but resistance to DDT is widespread among many efficient mosquito vectors in different parts of India [6–8, 29–31]. The KDT_50_ and KDT_99_ values were also found to be very high and never recorded below 91.4 minutes (95% CI = 79.8–109.3), suggesting that the tested mosquitoes were not much knock-down sensitive to DDT. However, relatively low value of KDT has been recorded in the regions where mosquitoes are susceptible to DDT, whereas high KDT values have been recorded from the regions which reported high level of DDT resistance [6, 29, 32]. DDT is most accepted insecticide in India and its use for many decades now has resulted in high selection pressure and widespread of insecticide resistance. Although *kdr* mutations have been reported to confer resistance to DDT, but a recent study has again raised this concern by suggesting that about 30% of phenotypically resistant mosquitoes did not present *kdr* mutations [33].

There was complete susceptibility to deltamethrin as the corrected mortalities recorded were above 98.1% for both the mosquito species in both the study areas. However a reduced sensitivity was observed for lambda-cyhalothrin in *An*. *vagus* as the corrected mortality observed ranged from 88.6 (95% CI = 84.9–91.9) to 97.3% (95% CI = 93.3–98.9). The KDT_50_ and KDT_99_ values for deltamethrin and lambda-cyhalothrin were comparable for both the species, but interestingly these values for lambda-cyhalothrin in *An*. *vagus* were found to be 1.5 (KDT_50_) and 1.7 (KDT_99_) fold high than deltamethrin in BPHC area.

Synthetic pyrethroids including deltamethrin and lambda-cyhalothrin are widely used in various public health programmes to control mosquitoes in many countries. However, in the recent years efficacy of these insecticides against potential malaria vectors has been found to reduced in endemic areas [2, 3, 6, 17, 34]. The present study area has vast paddy fields and large scale vegetable cultivation throughout the year, and the pyrethroids are widely used in the control of agricultural pests. Furthermore, the pyrethroid based long lasting insecticidal nets (LLINs) have been considered as the cornerstone of malaria control programmes and distributed free of cost by the government agencies in the recent years. All these activities have increased the selection in malaria vectors to this class of insecticides. A significant level of resistance to pyrethroid was found associated with the agriculture intensity in Africa, indicating that resistance level increases with the increase in agriculture spread [2, 35]. Presently, the tested mosquito were knock-down sensitive to pyrethroid as the KDT_50_ an KDT_99_ values were considerably lower but found to be higher than achieved for *An*. *annularis* previously [6]. In a study conducted in Mekong region, *An*. *vagus* was found to be highly knock-down resistant to deltamethrin and revealed the presence of a L1014S *kdr* mutation [17].

Present results revealed complete susceptibility to malathion except for *An*. *vagus* in BPHC area where the mortality recorded was 97.6% (95% CI = 95.4–98.8) indicating reduced susceptibility which warrants regular monitoring. Although malathion is extensively used in the control of vector mosquitoes in different endemic regions of India but no study has clearly indicated the development of resistance to malathion [29]. Malathion is mostly used in fogging to control dengue vectors during the epidemics and not in the control of malaria vectors, therefore the chances of exposing *Anopheles* mosquitoes to malathion are limited except some accidental exposure.

Mutations in the voltage gate sodium channel gene have been shown as important mechanism conferring high level of cross resistance to DDT and synthetic pyrethroids. Currently no evidence of cross resistance to DDT and pyrethroids has been observed, however the study has underlined the existence of DDT resistance in malaria vectors and possible decline in sensitivity to the synthetic pyrethroids, but does not suggests the mechanism which could be attributed to the problem of resistance. Studies have very well documented the role of target-site mutations in insecticide resistance, however these were not found solely responsible for resistance and some detoxifying genes acting in concert with these mutations in voltage gated sodium channels were reported to confer extreme levels of resistance [28, 35–39]. A recent research demonstrated that glutathione S-transferase gene GSTe2 was the most over-expressed detoxification gene in DDT and permethrin resistant *Anopheles funestus* mosquitoes [40], whereas another study [41] claimed that mutation L1014F was more efficient in conferring resistance to DDT as compared to pyrethroids, which might be a reason that the mosquitoes in the present study displayed high level of resistance to DDT. Furthermore, the studies have also suggested that *kdr* may act with certain unidentified co-factors to create resistance phenotype [42] or the resistance could be a multigenic phenomenon [43], thereby unable to fully explain the resistance mechanism. A study conducted in Bihar state of India to assess the utility of DDT based indoor residual spray found that sand flies were susceptible to deltamethrin but high level of resistance was observed to DDT [44].

In the present study, altogether five pools out of total 98 pools were detected positive using VectorTest^™^ for the presence of *Plasmodium* antigen suggesting that both these species play important role in malaria transmission. Four pools of *An*. *vagus* were found positive for *Plasmodium falciparum* revealing that *An*. *vagus* might be playing crucial role as malaria vector in the study area. During the past few years *An*. *vagus* has emerged as an important malaria vector, reported in large number in India and neighbouring countries [10,12] and incriminated as vector of malaria in India [45] and Bangladesh [12, 13]. *An*. *annularis* s.l. although found positive for *Plasmodium* in only one pool but regarded as important malaria vector in many endemic regions of India [6, 9]. In the present study primary malaria vectors *An*. *minimus* and *An*. *dirus* did not encounter, however recognised malaria vectors *An*. *culicifacies* (N = 68) and *An*. *fluviatilis* (N = 14) were recorded in low density and only one pool belonging to *An*. *culicifacies* s.l. in BPHC was found positive for *Plasmodium falciparum* sporozoite (MIR = 1.6). A recent investigation conducted in northeast India has revealed that 21.1% of the wild collected *An*. *annularis* were fed on human blood, while 2.6% were found positive for *Plasmodium falciparum* malaria parasite [6]. VectorTest^™^ antigen panel assay has been found effective in monitoring the disease spread by detecting malaria parasite in the wild collected mosquitoes [24, 25]. The used assay is rapid, one step procedure and qualitatively identifies specific peptide epitopes of circumsporozoites of the types of *Plasmodium* sporozoites.

The present results confirm the resistance to DDT and reduced susceptibility of pyrethroid insecticides which could gradually increase and spread into the other areas where complete susceptibility is reported at present. Mosquito control research and comprehensive vector tool development requires thorough analysis of such results and their consequences in a large area. Current study was carried out in two high malaria reporting and logistically accessible areas, however such studies using other malaria vectors should also be conducted in far flung and inaccessible areas which experience considerable toll of malaria related mortality and morbidity annually. Large number of mosquito specimen corresponding to well known and all possible malaria vector species are needed to be tested from different areas to get a clear insight about the role of each vector in malaria transmission in northeast India.

## Conclusion

For the first time field collected *An*. *vagus* and *An*. *annularis* s.l. mosquitoes using such a large sample size were evaluated against different insecticides in northeast India and found completely resistant to DDT, while completely sensitive to deltamethrin. Lambda-cyhalothrin susceptibility was reduced in *An*. *vagus*. Further investigations are recommended to understand the mechanism underlying the phenotypical resistance to DDT and declining susceptibility to synthetic pyrethroid in order to guide judicious selection of suitable insecticides for vector control interventions. Resistance management strategies should be identified and considered to delay the expansion of insecticide resistance. Present results strongly advocate that both *An*. *annularis* and *An*. *vagus* may be playing more important role in malaria transmission than thought previously. Testing of *Plasmodium* parasite presence in the field caught potential *Anopheles* vectors prevailing in high density from different areas, in addition to the well established vectors, could be useful to highlight the role of these little known and practically ignored vectors in malaria transmission.

## Supporting Information

S1 Table knock-down of *An*. *annularis* and *An*. *vagus* in OPHC. (XLS)

S2 Table Knock-down of *An*. *annularis* and *An*. *vagus* mosquitoes in BPHC. (XLS)

S3 Table Data of Vectortest malaria sporozoite panel assay. (XLS)
